# Cardiac effects and comorbidities of neurological diseases

**DOI:** 10.55730/1300-0144.5928

**Published:** 2024-10-31

**Authors:** Bilgin ÖZTÜRK

**Affiliations:** Neurology Department, Gülhane Training and Research Hospital, University of Health Sciences, Ankara, Turkiye

**Keywords:** Cardiovascular complications, stroke, epilepsy, Parkinson’s disease, multiple sclerosis, dementia

## Abstract

Neurological disorders encompass a complex and heterogeneous spectrum of diseases affecting the brain, spinal cord, and peripheral nervous system, each presenting unique challenges that extend well beyond primary neurological symptoms. These disorders profoundly impact cardiovascular health, prompting an intensified exploration into the intricate interconnections between the neurological and cardiovascular systems. This review synthesizes current insights and research on cardiovascular comorbidities associated with major neurological conditions, including stroke, epilepsy, Parkinson’s disease, multiple sclerosis, and Alzheimer’s disease. The cardiovascular sequelae of these neurological disorders are multifactorial. For instance, strokes not only predispose individuals to arrhythmia and heart failure but also exacerbate preexisting cardiovascular risk factors. Similarly, epilepsy is associated with autonomic dysregulation and an elevated risk of sudden cardiac death, underscoring the necessity for vigilant cardiac monitoring in affected individuals. Parkinson’s disease manifests with orthostatic hypotension and cardiac sympathetic denervation, significantly contributing to morbidity. Additionally, multiple sclerosis and Alzheimer’s disease exhibit cardiovascular autonomic dysfunction and heightened cardiovascular risk, underscoring the need for proactive management strategies.

Mechanistically, these conditions disrupt autonomic nervous system regulation, induce chronic inflammation, and may share genetic susceptibilities, each contributing to cardiovascular pathology. Effective management of these complexities requires an integrative approach that includes risk factor modification, pharmacotherapy, lifestyle interventions, and comprehensive patient education.

Future research directions include identifying novel therapeutic targets, conducting large-scale clinical trials, and investigating genetic biomarkers to individualize treatment strategies. By addressing the multifaceted interactions between neurological disorders and cardiovascular health, healthcare providers can optimize patient care, reducing cardiovascular morbidity and mortality in this vulnerable population.

## 1. Introduction

Neurological diseases encompass a broad spectrum of disorders affecting the brain, spinal cord, and peripheral nerves. These conditions not only manifest with primary neurological symptoms such as cognitive impairment, motor dysfunction, and sensory deficits but also exert profound secondary effects on the cardiovascular system [1–5]. Several studies on the neurological effects of COVID, especially during the COVID pandemic, reminded us that systemic or infectious diseases may have neurological effects [6–8]. It is known that many heart-related diseases or conditions affect the nutrition of the brain and, accordingly, its proper functioning [9]. The intricate relationship between neurological and cardiac health underscores the importance of comprehensive management strategies to optimize patient care and improve outcomes.

This review aims to provide a detailed exploration of the cardiac effects associated with major neurological diseases, including stroke, epilepsy, Parkinson’s disease, multiple sclerosis (MS), and Alzheimer’s disease (AD). Each of these conditions presents unique challenges and implications for cardiovascular health, ranging from increased risk of arrhythmias and stroke to potential impacts on heart function and vascular integrity.

By examining the underlying mechanisms linking neurological and cardiovascular systems, the aim of this paper is to elucidate the pathophysiological connections and clinical implications. Furthermore, the review will highlight integrated care approaches that integrate neurological and cardiac perspectives, aiming to optimize diagnostic accuracy, therapeutic efficacy, and patient outcomes.

The investigation of cardiac manifestations in neurological diseases has evolved significantly from early clinical observations to modern scientific inquiry. Historically, the focus was primarily on unraveling the neurological aspects of these conditions, often overlooking or underestimating their profound impact on cardiac health. However, advancements in medical imaging, neurophysiology, and cardiovascular medicine have illuminated the intricate relationship between neurological disorders and cardiovascular dysfunction.

Studies identified shared risk factors and pathophysiological mechanisms between neurological and cardiovascular diseases [2,7,8,10–17]. These foundational insights catalyzed further research into autonomic nervous system dysregulation, neuroinflammation, genetic predispositions, and the cardiac effects of medications used in treating neurological disorders.

The recognition of autonomic nervous system dysregulation as a critical link between neurological and cardiovascular systems has been particularly significant. Dysautonomia contributes to a range of cardiac complications, including arrhythmia, orthostatic hypotension, and altered heart rate variability, thereby impacting the overall prognosis and quality of life for patients with neurological conditions.

Moreover, the role of neuroinflammation in promoting vascular endothelial dysfunction and atherosclerosis underscores the systemic nature of these diseases. Genetic studies have identified shared susceptibility genes influencing both neurological and cardiovascular disorders, highlighting potential targets for future therapeutic interventions aimed at mitigating disease progression.

Furthermore, the cardiac effects of neuropharmacological treatments, such as antiepileptic drugs and dopaminergic agents, necessitate careful consideration in clinical practice. These medications can affect cardiac electrophysiology, myocardial contractility, and vascular tone, necessitating integrated care approaches that address both neurological and cardiovascular health.

In summary, the evolving understanding of cardiac manifestations in neurological diseases underscores the importance of interdisciplinary collaboration between neurology and cardiology. By integrating insights from both fields, healthcare professionals can optimize diagnostic accuracy, therapeutic strategies, and patient outcomes in this complex patient population.

## 2. Cardiac effects of neurological diseases

Neurological diseases can profoundly affect cardiac function through diverse mechanisms, leading to a spectrum of cardiovascular complications. These conditions, which encompass disorders affecting the brain, spinal cord, and peripheral nerves, often involve disruptions in autonomic nervous system regulation [18]. Such dysregulation can manifest as arrhythmias, alterations in heart rate variability, and impaired baroreflex sensitivity. Additionally, neuroinflammatory processes, oxidative stress, and the effects of neuropharmacological treatments further contribute to cardiovascular morbidity in patients with neurological disorders [15,19]. Understanding these complex interactions is crucial for developing targeted management strategies that address both neurological and cardiovascular health, thereby improving overall patient outcomes and quality of life. This section provides an in-depth exploration of the cardiac effects associated with each major neurological disease.

### 2.1. Stroke

Stroke, encompassing both ischemic and hemorrhagic types, remains a predominant cause of global morbidity and mortality. Beyond its immediate neurological impact, stroke can induce significant cardiovascular disturbances that profoundly affect patient outcomes and management strategies.

One of the critical cardiovascular complications associated with stroke is the development of arrhythmias, notably atrial fibrillation, during the acute phase [2,18]. Atrial fibrillation increases the risk of thromboembolic events, including stroke recurrence, necessitating vigilant monitoring and often long-term anticoagulation therapy to mitigate these risks effectively. Atrial fibrillation and other cardiac diseases are widely accepted as significant contributors to ischemic stroke risk. However, after a stroke (especially in the presence of other chronic diseases such as diabetes), arrhythmias may also develop due to cardiac damage [20]. Furthermore, stroke-related damage to the myocardium or neurohormonal disturbances can precipitate acute or chronic heart failure [2]. Although it is clear that atrial fibrillation causes ischemic stroke, the cause–effect relationship between ischemic stroke and cardiac arrhythmias is less clear [21]. The interplay between neurological insults and cardiac dysfunction poses substantial challenges in patient care, requiring integrated approaches to optimize cardiovascular and neurological outcomes simultaneously.

Ischemic heart disease represents another significant concern for stroke survivors, exacerbated by shared risk factors such as hypertension, diabetes mellitus, and dyslipidemia [3]. Effective management of these modifiable risk factors is essential in reducing the incidence of secondary cardiovascular events and improving long-term prognosis.

Recent studies highlight the bidirectional relationship between stroke and cardiac dysfunction, underscoring the importance of integrated therapeutic strategies. Comprehensive care that addresses both neurological rehabilitation and cardiovascular risk management is crucial for enhancing patient recovery and minimizing complications.

In conclusion, the complex interactions between stroke and cardiovascular health necessitate holistic approaches that integrate neurological and cardiac care. By understanding and addressing shared risk factors, monitoring cardiovascular complications, and implementing evidence-based interventions, healthcare providers can significantly improve outcomes for stroke patients, thereby reducing morbidity and mortality associated with these devastating events.

### 2.2. Epilepsy

Epilepsy, characterized by recurrent seizures, is associated with significant cardiovascular implications that extend beyond its neurological manifestations. Individuals with epilepsy face a 2–3 times greater risk of early death compared to the general population, with approximately 15% of these premature deaths resulting from sudden cardiac death or acute myocardial infarctions. Moreover, the length of epilepsy with recurrent seizures is probably associated with the progressive development of cardiac damage [22]. Sudden unexpected death in epilepsy (SUDEP) represents a substantial cause of mortality among epilepsy patients and is often associated with cardiac arrhythmias occurring during or immediately after seizures [4]. The sudden and unpredictable nature of seizures can precipitate cardiac dysrhythmias, including ventricular tachycardia or fibrillation, leading to fatal outcomes in vulnerable individuals. Seizures disrupt the autonomic nervous system’s regulation of heart rate, blood pressure, and vascular tone, resulting in profound cardiovascular effects [10,12,23]. These disruptions can manifest as bradyarrhythmia, such as sinus bradycardia or atrioventricular block, as well as tachyarrhythmias like supraventricular tachycardia [5]. Elevated levels of T waves are associated with an increased risk of lethal arrhythmias. So-called “epileptic heart” has been proposed by Verrier and colleagues as “a heart and coronary vasculature damaged by chronic epilepsy as a result of repeated surges in catecholamines and hypoxemia leading to electrical and mechanical dysfunction” [24]. The variability in autonomic response during and after seizures poses challenges in managing cardiovascular stability in epilepsy patients [25]. Beyond acute arrhythmias, epilepsy patients often exhibit an increased prevalence of traditional cardiovascular risk factors such as hypertension, dyslipidemia, and obesity [11]. Sodium channel-blocking agents especially at high doses in combination with other sodium channel blocking drugs were reported to cause cardiac arrhythmias even in the absence of preexisting cardiac diseases [22]. The chronic use of antiepileptic drugs (AEDs), particularly older generation medications like phenytoin or carbamazepine, may further contribute to cardiovascular complications through mechanisms such as QT prolongation or metabolic disturbances [26].

Managing epilepsy requires a comprehensive approach that integrates neurological and cardiovascular considerations, particularly in treatment-resistant cases or those with significant comorbidities. Patients’ history should be carefully evaluated for risk factors such as congenital heart disease, arrhythmia, dyspnea, chest pain, hypertension, hyperlipidemia, diabetes mellitus, smoking, and any family history of any cardiovascular diseases [27]. Regular cardiovascular assessments, including electrocardiographic monitoring and periodic echocardiography, can aid in detecting and managing cardiac abnormalities early. Optimizing AED selection to minimize cardiovascular side effects and implementing lifestyle modifications to mitigate cardiovascular risk factors are essential components of comprehensive epilepsy care. By addressing both the neurological and cardiovascular aspects of epilepsy, healthcare providers can enhance patient safety, improve seizure control, and reduce the overall cardiovascular burden in this vulnerable population. People with epilepsy who are at risk for any cardiac complications should be referred to a cardiologist.

### 2.3. Parkinson’s disease

Parkinson’s disease, a progressive neurodegenerative disorder, presents with a spectrum of symptoms that extend beyond its well-known motor impairments. Among its nonmotor symptoms, significant autonomic dysfunction plays a crucial role in shaping its cardiovascular manifestations. Since Parkinson’s disease, like cardiac diseases, increases in prevalence with age, the risk of comorbidity with cardiac diseases is higher. Aging has been shown to be a significant risk factor for both cardiovascular illnesses and Parkinson’s disease [28]. The most common cardiovascular abnormalities in Parkinson’s patients are related to dysautonomia, while structural heart diseases are less common. However, previous research has shown a positive link between cardiovascular diseases and Parkinson’s disease [29].

Autonomic nervous system dysfunction is accepted as a nonmotor involvement in Parkinson’s disease that occurs long before the appearance of standard motor signs and symptoms [15,30]. One of the prominent cardiovascular complications in Parkinson’s disease is orthostatic hypotension. This hypotension can occur even orthostatic or postprandial hypotension, either nocturnal or supine hypertension [31]. This condition arises from dysfunction in the autonomic nervous system, leading to an inadequate adjustment of blood pressure upon standing. Orthostatic hypotension not only increases the risk of falls and syncope but also poses daily challenges for patients, affecting their mobility and quality of life.

Another critical aspect of cardiac involvement in Parkinson’s disease is cardiac sympathetic denervation [32]. The degeneration of sympathetic nerves within the heart results in diminished heart rate variability and an increased susceptibility to arrhythmias. This dysregulation complicates the management of cardiovascular health in Parkinson’s disease patients, requiring careful monitoring and intervention to mitigate risks.

Patients with Parkinson’s disease are at risk of developing heart disease as a side effect of their medication. Levodopa can exacerbate orthostatic hypotension, while dopamine agonists can cause restrictive valvular heart disease [33,34].

Effective management of Parkinson’s disease necessitates a comprehensive approach that integrates the treatment of both motor and nonmotor symptoms, including autonomic dysfunction. Strategies to address cardiovascular complications involve lifestyle modifications such as adequate hydration and gradual postural changes to manage orthostatic hypotension. Pharmacological interventions targeting blood pressure regulation and heart rate variability may also be applied under medical supervision.

Furthermore, the optimization of Parkinson’s disease therapies to minimize adverse effects on cardiovascular function is crucial. This includes selecting medications that have minimal impact on heart rhythm and function. Collaborative care involving neurologists, cardiologists, and other healthcare professionals is essential for tailored management plans that consider the complex interactions between neurological and cardiovascular health in Parkinson’s disease.

### 2.4. Multiple sclerosis

MS is an autoimmune disorder affecting the central nervous system. Since MS is seen in relatively young people, its cardiac effects are less significant than those of other neurological diseases (Parkinson’s disease, stroke, etc.) [35]. However, MS can significantly impact cardiovascular health through various mechanisms. MS-related lesions, especially in the brainstem, disrupt central autonomic pathways, leading to abnormalities in heart rate variability and blood pressure regulation. This autonomic dysfunction can manifest as orthostatic hypotension, arrhythmias, and impaired baroreflex sensitivity, affecting cardiovascular stability and increasing the risk of adverse cardiac events in MS patients. Chronic inflammation associated with MS, compounded by physical disability and sedentary lifestyle, contributes to an elevated risk of cardiovascular diseases. Conditions such as stroke and myocardial infarction might be more prevalent in MS patients than in the general population due to immobilization in the late period of the disease, highlighting the need for proactive cardiovascular risk management strategies [36]. Autonomic dysfunctions appear to be associated with disease duration and to be more common in progressive forms of MS [37].

MS patients also showed impaired biventricular function with reduced LA function, but normal arterial and endothelial function [14].

The comprehensive care of MS patients requires an integrated approach that addresses both neurological and cardiovascular aspects. Lifestyle modifications, including regular physical activity and dietary adjustments, are crucial for managing cardiovascular risk factors such as hypertension, hyperlipidemia, and obesity in MS patients. Regular cardiac assessments, including electrocardiography and echocardiography, can aid in early detection of cardiac abnormalities and guide timely interventions [14,36,37]. Selecting MS treatments that minimize cardiovascular side effects is essential [38,39]. Monitoring potential cardiotoxic effects of certain disease-modifying therapies is critical in managing both MS progression and cardiovascular health.

### 2.5. Alzheimer’s disease

AD, the most prevalent form of dementia, is increasingly recognized for its significant cardiovascular implications.

AD shares common risk factors such as hypertension, diabetes mellitus, and hyperlipidemia with cardiovascular diseases [40]. These risk factors contribute to a higher incidence of cardiovascular conditions such as coronary artery disease, heart failure, and atrial fibrillation in individuals with AD. The presence of these comorbidities complicates the management of both cognitive and cardiovascular health in affected patients.

Vascular pathology plays a crucial role in exacerbating cognitive decline in AD patients. Conditions such as atherosclerosis and cerebral small vessel disease contribute to microvascular damage and ischemic events, which further impair cognitive function and increase the risk of ischemic stroke [17]. The interplay between vascular pathology and neurodegeneration in AD underscores the importance of addressing both aspects for comprehensive disease management.

Early detection and effective management of cardiovascular risk factors in AD are critical for optimizing cognitive and cardiovascular outcomes. Routine screening for hypertension, diabetes, and dyslipidemia is essential in AD patients to identify and manage cardiovascular risk factors early. This includes monitoring blood pressure, glucose levels, and lipid profiles to prevent or delay the onset of cardiovascular complications. Encouraging healthy lifestyle habits such as regular physical activity, a balanced diet, smoking cessation, and weight management can help mitigate cardiovascular risk factors and potentially slow the progression of cognitive decline in AD.

Depending on individual risk profiles, pharmacological interventions may be necessary to control hypertension, diabetes, and dyslipidemia in AD patients. Careful consideration of medication interactions and potential cognitive effects is crucial in selecting appropriate treatments.

By addressing cardiovascular risk factors early and comprehensively in individuals with AD, healthcare providers can potentially improve both cognitive and cardiovascular outcomes. Integrated care approaches that consider the complex relationship between AD and cardiovascular health are essential for enhancing overall quality of life and reducing the burden of disease in affected individuals and their caregivers.

## 3. Mechanisms linking neurological diseases to cardiac effects

The cardiovascular complications observed in neurological diseases arise from intricate and interconnected mechanisms, underscoring the complex relationship between neurological and cardiac health.

### 3.1. Autonomic nervous system dysregulation

Neurological diseases frequently disrupt the autonomic nervous system (ANS), which plays a pivotal role in regulating cardiovascular function.

Conditions such as stroke and epilepsy can precipitate sympathetic overactivity, characterized by heightened sympathetic tone leading to increased heart rate, blood pressure variability, and predisposition to arrhythmias [10,15,17,18,23,29,41]. The dysregulated sympathetic response exacerbates cardiovascular instability and poses significant management challenges in neurological patients.

Disorders like Parkinson’s disease and MS often impair parasympathetic control, resulting in reduced heart rate variability and predisposition to bradyarrhythmia. This dysfunction in vagal tone complicates the cardiovascular management of these patients, necessitating tailored therapeutic approaches to mitigate cardiac risks.

Understanding ANS dysregulation is essential for devising effective therapeutic interventions aimed at mitigating cardiac complications in neurological patients.

### 3.2. Inflammation

Chronic inflammation is a hallmark feature of many neurological diseases and plays a pivotal role in driving cardiovascular pathology.

Inflammatory cytokines, such as interleukin-6 (IL-6) and tumor necrosis factor-alpha (TNF-alpha), promote endothelial dysfunction and accelerate atherosclerotic processes in cerebral and systemic vasculature [19]. This chronic inflammatory state increases the risk of developing ischemic heart disease and stroke in patients with neurological disorders. The synergistic effects of neuroinflammation and vascular endothelial damage contribute to a heightened susceptibility to cardiovascular events.

Direct effects of inflammatory mediators on myocardial tissue, including oxidative stress and fibrosis, can impair contractility and contribute to the development of heart failure [42,43]. The systemic inflammatory burden on neurological diseases underscores the potential role of antiinflammatory therapies in managing cardiac complications and improving overall cardiovascular outcomes in affected individuals. Targeting inflammation represents a promising therapeutic strategy for mitigating cardiovascular risk in neurological patients, necessitating integrated approaches in disease management that address both neurological and cardiac aspects.

### 3.3. Genetic factors

Genetic predispositions influence susceptibility to both neurological and cardiovascular diseases. Mutations affecting lipid metabolism (e.g., ApoE genotype), ion channels (e.g., SCN5A mutations), and inflammatory pathways (e.g., TNF-alpha polymorphisms) predispose individuals to conditions such as familial hypercholesterolemia and familial amyloid polyneuropathy, which manifest with neurological and cardiac involvement [44,45]. These shared genetic pathways highlight the intricate interplay between neurological and cardiovascular health and underscore the importance of genetic screening in identifying at-risk individuals.

Identifying genetic markers may facilitate early detection and personalized management strategies in affected individuals, aiming to mitigate the impact of both neurological and cardiac manifestations and improve long-term outcomes.

### 3.4. Medication side effects

Pharmacological treatments for neurological diseases are essential for managing symptoms but can also carry significant cardiovascular risks that healthcare providers must carefully navigate. For instance, antiepileptic drugs like carbamazepine and phenytoin are known to prolong the QT interval, potentially increasing the risk of ventricular arrhythmias and sudden cardiac death [10,26]. These treatments are widely used to control seizures, yet their impact on cardiac function underscores the importance of regular monitoring and assessment to mitigate cardiovascular complications.

In addition to antiepileptics, antipsychotic medications such as haloperidol and risperidone are commonly prescribed to manage psychiatric symptoms in neurological disorders. However, these drugs can induce metabolic changes and prolong the QT interval, which poses a significant concern, particularly in patients with existing cardiovascular conditions [46]. The careful management of these side effects is crucial to minimize the risk of adverse cardiac events while maximizing the therapeutic benefits of these medications in improving neurological symptoms.

Balancing the therapeutic benefits with potential cardiovascular risks requires a personalized approach tailored to each patient’s specific neurological and cardiovascular profile. Healthcare providers must collaborate closely with specialists in cardiology and neurology to develop comprehensive treatment plans that address both the neurological and cardiac aspects of patient care. By integrating regular cardiovascular assessments, monitoring for QT interval prolongation, and adjusting medication regimens as needed, healthcare teams can optimize patient safety and enhance overall treatment outcomes in individuals with neurological diseases.

## 4. Diagnosis and monitoring of cardiac effects in neurological patients

Early detection and meticulous monitoring of cardiac effects in neurological patients are pivotal for optimizing clinical outcomes and enhancing overall quality of care. Comprehensive cardiovascular screening is essential, encompassing regular assessments of traditional risk factors such as hypertension, diabetes mellitus, and dyslipidemia, alongside performing electrocardiography (ECG) and echocardiography. These diagnostic modalities not only aid in the early identification of subclinical cardiac dysfunction but also provide valuable insights into the structural and electrical aspects of the heart, guiding timely therapeutic interventions to mitigate cardiovascular risks.

Moreover, Holter monitoring represents a valuable adjunct in the diagnostic armamentarium, offering continuous ambulatory ECG monitoring to detect arrhythmias and evaluate heart rate variability. This approach is particularly beneficial for patients suspected of autonomic dysfunction, where subtle changes in cardiac function may manifest unpredictably during daily activities. The data gathered from Holter monitoring enables healthcare providers to capture transient cardiac abnormalities that may escape detection during routine clinical visits, facilitating more accurate diagnosis and informed management decisions.

Integrating comprehensive cardiac monitoring into routine neurological care practices enhances the capacity for early intervention and risk stratification, thereby optimizing patient management strategies. By proactively identifying cardiac issues at their inception, healthcare teams can implement targeted interventions tailored to each patient’s unique cardiovascular profile. This proactive approach not only mitigates the potential for adverse cardiovascular events but also improves long-term outcomes and enhances the overall well-being of neurological patients.

Furthermore, fostering a multidisciplinary approach involving neurologists, cardiologists, and other allied healthcare professionals is crucial. This collaborative effort ensures that neurological patients with underlying or emerging cardiac concerns receive integrated and holistic care, addressing both neurological and cardiovascular aspects comprehensively. By leveraging advanced diagnostic tools and a coordinated care approach, healthcare providers can uphold the highest standards of patient-centered care and achieve optimal outcomes for individuals navigating the complex interplay between neurological and cardiac health.

## 5. Management and treatment approaches

Neurologists play a crucial role in the initial assessment and ongoing management of neurological conditions, focusing on disease-specific therapies to mitigate neurological symptoms and progression. However, the potential cardiovascular implications of these diseases require collaboration with cardiologists and other cardiovascular specialists. This collaborative approach ensures thorough cardiovascular risk assessment and management tailored to the individual patient’s needs. Managing cardiovascular complications in neurological patients requires a multidisciplinary approach that addresses both neurological and cardiac aspects.

### 5.1. Pharmacological interventions

Pharmacological interventions are essential in the multidisciplinary management of cardiovascular complications in neurological patients, addressing specific cardiovascular risks associated with various neurological diseases. Beta-blockers, such as metoprolol and propranolol, are commonly used to regulate heart rate and blood pressure, particularly in patients at risk of arrhythmias and hypertension. Their role extends to stabilizing cardiac function in conditions like Parkinson’s disease, where autonomic dysfunction can lead to fluctuating blood pressure and heart rate [4,34].

Furthermore, anticoagulants such as warfarin and direct oral anticoagulants are crucial in preventing thromboembolic events in patients with neurological diseases complicated by atrial fibrillation. Stroke, a common sequela of neurological disorders like AD and stroke itself, necessitates effective anticoagulation to reduce the risk of recurrent ischemic events and improve long-term prognosis. These medications are integral to secondary stroke prevention strategies, highlighting their essential role in managing cerebrovascular complications.

Incorporating these pharmacological interventions into the treatment regimen of neurological patients requires close collaboration between neurologists, cardiologists, and other healthcare professionals. This collaborative approach ensures comprehensive cardiovascular risk assessment, personalized treatment plans, and ongoing monitoring to optimize patient outcomes and enhance quality of life amidst the complex intersection of neurological and cardiovascular health.

### 5.2. Lifestyle modifications

Nonpharmacological interventions are integral components of managing cardiovascular complications in neurological patients, focusing on lifestyle modifications that complement pharmacological treatments and improve overall health outcomes.

Promoting a heart-healthy diet low in saturated fats and sodium is crucial for managing hypertension and dyslipidemia in neurological patients. Emphasizing the consumption of fruits, vegetables, whole grains, and lean proteins can help control blood pressure and cholesterol levels, reducing the risk of cardiovascular events. This dietary approach is particularly beneficial in conditions such as multiple sclerosis and AD, where chronic inflammation and metabolic changes contribute to cardiovascular risk.

Tailored exercise regimens play a pivotal role in improving cardiovascular fitness and reducing the risk of stroke recurrence in neurological patients. Regular physical activity enhances vascular health, promotes weight management, and improves overall well-being. It is essential in conditions like Parkinson’s disease and stroke rehabilitation, where maintaining mobility and functional independence are key goals of treatment. Exercise programs should be individualized based on the patient’s neurological condition, functional abilities, and cardiovascular risk profile to optimize benefits while minimizing potential risks.

Counseling and pharmacotherapy interventions are critical in assisting neurological patients to quit smoking, as tobacco use significantly increases cardiovascular risk. Smoking cessation not only reduces the incidence of stroke and cardiovascular disease but also enhances the effectiveness of pharmacological treatments and improves overall treatment outcomes. Addressing tobacco dependence is essential across all neurological disorders to mitigate the detrimental effects of smoking on both neurological and cardiovascular health.

Integrating these nonpharmacological interventions into the comprehensive care plan for neurological patients requires a collaborative approach involving neurologists, dietitians, physiotherapists, and smoking cessation specialists. By promoting healthy lifestyle habits, healthcare providers can enhance cardiovascular resilience, optimize treatment efficacy, and ultimately improve the long-term prognosis and quality of life for individuals navigating the complexities of neurological and cardiovascular comorbidities.

### 5.3. Preventive strategies

Risk factor management is paramount in the holistic approach to preventing cardiovascular events in neurological patients, requiring proactive strategies to control hypertension, diabetes mellitus, and dyslipidemia. Effective management of these risk factors not only reduces the incidence of cardiovascular complications but also mitigates the progression of neurological diseases and improves overall patient outcomes.

Aggressive control of hypertension is crucial, as elevated blood pressure contributes significantly to the risk of stroke and cardiovascular events in conditions like MS and Parkinson’s disease [2,18,27,28]. Early identification through regular blood pressure monitoring and prompt initiation of antihypertensive therapies tailored to individual patient needs are essential steps in reducing vascular complications.

Similarly, optimizing management of diabetes mellitus is imperative, given its association with accelerated atherosclerosis and increased cardiovascular morbidity in neurological patients [2]. Close monitoring of blood glucose levels, adherence to prescribed medications (e.g., insulin or oral hypoglycemic agents), and lifestyle modifications (e.g., diet and exercise) are fundamental in controlling diabetes and minimizing its impact on cardiovascular health. Dyslipidemia management involves targeting elevated cholesterol levels with statins and lifestyle modifications aimed at reducing dietary intake of saturated fats and cholesterol [2]. This approach is critical in reducing the risk of atherosclerotic cardiovascular disease, which is prevalent in neurological conditions such as AD and stroke [2,17].

Patient education plays a pivotal role in promoting adherence to medications and lifestyle modifications essential for managing cardiovascular risk factors. Educating patients about the importance of medication compliance, dietary choices, regular exercise, and smoking cessation empowers them to actively participate in their care, fostering better cardiovascular outcomes and overall health.

Multidisciplinary care, involving collaboration among neurologists, cardiologists, primary care physicians, and other healthcare professionals, ensures comprehensive assessment and management of both neurological and cardiac aspects. This collaborative approach facilitates early detection of cardiovascular complications, personalized treatment plans, and coordinated care delivery, thereby optimizing patient outcomes and enhancing quality of life.

By integrating rigorous risk factor management, patient education initiatives, and multidisciplinary care models into clinical practice, healthcare providers can effectively mitigate cardiovascular risks in neurological patients. This comprehensive approach not only addresses immediate health concerns but also promotes long-term cardiovascular health and overall well-being in individuals with complex neurological conditions.

## 6. Future directions and research

Future research endeavors should focus on advancing our understanding of the complex interactions between neurological diseases and cardiac health.

Further elucidation of the molecular pathways linking neurological disorders to cardiovascular dysfunction is crucial for developing targeted therapies that address underlying pathophysiology.

Large-scale trials evaluating the efficacy of novel therapeutic agents and interventions in preventing and managing cardiovascular complications in neurological patients are warranted to establish evidence-based guidelines.

Identification of genetic biomarkers associated with cardiovascular risk in neurological diseases may facilitate early risk stratification and personalized treatment approaches.

Advancements in these areas hold promises for improving diagnostic accuracy, treatment efficacy, and patient outcomes in the realm of neurological and cardiac comorbidities.

## 7. Conclusion

The intricate interplay between neurological diseases and cardiac health underscores the critical importance of integrated care approaches in clinical practice. Across conditions such as stroke, epilepsy, Parkinson’s disease, MS, and AD, there exists a complex relationship where neurological manifestations often intertwine with significant cardiovascular implications. Each condition presents unique challenges and opportunities for proactive cardiovascular management, necessitating a comprehensive and tailored approach to patient care.

Shared risk factors play a pivotal role in driving cardiovascular complications in neurological patients. Hypertension, diabetes mellitus, and dyslipidemia are prevalent across various neurological disorders and contribute significantly to increased cardiovascular risk. Addressing these risk factors early and effectively through rigorous monitoring and targeted interventions is essential for mitigating adverse cardiovascular outcomes and improving overall patient prognosis.

Understanding the underlying mechanisms linking neurological and cardiac health provides critical insights into disease pathophysiology and therapeutic strategies. For example, autonomic nervous system dysregulation is a common feature in many neurological diseases, influencing heart rate variability and blood pressure regulation. Inflammation, another shared mechanism, exacerbates cardiovascular pathology through endothelial dysfunction and atherosclerosis, emphasizing the need for antiinflammatory therapies in disease management.

Multidisciplinary collaboration is fundamental in optimizing patient outcomes. Neurologists, cardiologists, primary care physicians, and allied healthcare professionals work together to develop holistic treatment plans that address both neurological and cardiovascular aspects comprehensively. This collaborative approach ensures early detection of cardiovascular complications, personalized treatment strategies, and ongoing monitoring to adjust interventions as needed based on individual patient responses.

Continued advancements in research and clinical innovations are pivotal for advancing our understanding and management of cardiovascular risks in neurological diseases. Through ongoing studies into disease-specific mechanisms, biomarkers, and therapeutic interventions, clinicians can refine diagnostic approaches, enhance treatment efficacy, and ultimately improve long-term patient outcomes.

In conclusion, this article advocates for a holistic and integrated approach to managing cardiovascular complications in neurological patients. By addressing shared risk factors, understanding disease mechanisms, and fostering multidisciplinary care models, healthcare providers can optimize patient care, reduce the burden of cardiovascular disease, and improve the quality of life for individuals affected by these complex conditions. This proactive and comprehensive strategy underscores the transformative impact of collaborative healthcare in navigating the intricate interactions between neurological diseases and cardiac health.
